# One hundred years of neuropsychology: Kurt Goldstein’s seminal paper on the inherent complexity of symptoms

**DOI:** 10.1590/1980-5764-DN-2024-0255

**Published:** 2025-09-01

**Authors:** Stefan Frisch, Alexandre Métraux

**Affiliations:** 1 Pfalzklinikum for Psychiatry and Neurology, Department of Geriatric Psychiatry, Psychosomatic Medicine, and Psychotherapy, Klingenmünster, Rhineland-Palatinate, Germany.; 2 Université de Lorraine, Archives Henri Poincaré, Nancy, Grand Est, France.

**Keywords:** Kurt Goldstein, Alexander R. Luria, Neuropsychology, Signs and Symptoms, Kurt Goldstein, Alexander R. Luria, Neuropsicologia, Sinais e Sintomas

## Abstract

One year after the German-American neurologist and psychiatrist Kurt Goldstein had died, the Soviet-Russian neurologist Alexander R. Luria published a brief obituary-like article in which he praised Goldstein’s 1925 lecture *Das Symptom* (*The symptom*) as *the* founding text of neuropsychology. Some reasons given by Luria for praising Goldstein’s paper, however, seem unclear. The present contribution presents the core ideas of Goldstein’s paper, and analyses in which way Luria may not have fully grasped its scope. In fact, *Das Symptom* looks rather like a founding lecture on how to convincingly understand neuropathological symptoms for the benefit, among other things, of neuropsychology, with implications even for present-day neuroscience.

## RESUMO

Um ano após a morte do neurologista e psiquiatra germano-americano Kurt Goldstein, o neurologista soviético-russo Alexander R. Luria publicou um breve artigo semelhante a um obituário no qual ele elogiou a palestra de Goldstein de 1925, *Das Symptom* (*O sintoma*), como o texto fundador da neuropsicologia. As razões dadas por Luria para elogiar o artigo de Goldstein, no entanto, são em parte pouco claras. A presente contribuição analisa de que forma Luria não compreendeu totalmente seu escopo e em que medida *Das Symptom* parece ser de facto uma palestra fundadora sobre como compreender de forma convincente os sintomas neuropatológicos em benefício, entre outras coisas, da neuropsicologia, o que tem implicações até mesmo para a neurociência atual.

## INTRODUCTION

On June 7, 1925, the German neurologist and psychiatrist Kurt Goldstein (1878-1965) delivered a lecture at the fiftieth meeting of the South-Western German Association of Neurologists and Psychiatrists in Baden-Baden. The contribution was published one year later as a journal article entitled *Das Symptom, seine Entstehung und Bedeutung für unsere Auffassung vom Bau und von der Funktion des Nervensystems* ^1^ (The symptom, its origin and significance for our understanding of the architecture and functioning of the nervous system, our transl.; henceforth: *Das Symptom*). Almost thirty years later, the renowned Soviet neurologist and neuropsychologist Alexander R. Luria (1902-1977) referred to this article as “[…] the start of neuropsychology*”* (p. 312) in an obituary^2^ for Goldstein, who had died one year prior. Goldstein’s paper, which was never translated, was posthumously reprinted in his *Selected Papers* ^3^, which include 18 of his more than 300 publications. *Das Symptom* was written while the author practiced at Frankfurt on the Main as head of a military clinic for World War One brain wounded veterans as well as professor of neuropathology at Ludwig Edinger’s (1855-1918) famous *Neurological Institute* from 1914 to 1930. The Frankfurt years are presumably Goldstein’s most creative and productive period, during which he developed his organismic, “holistic” approach to neurology and neuropsychology, eventually summed up in his key monograph *Der Aufbau des Organismus* ^4^, before emigrating from Nazi Germany to the USA. Luria’s^2^ appreciation of *Das Symptom* is certainly correct, as it sums up Goldstein’s core ideas and introduces a novel perspective on neuropsychology as a whole. However, as will be demonstrated, Luria seems to have overlooked one core idea of *Das Symptom* and its continued relevance to neuropsychology and neuroscience today.

## VON MONAKOW’S CRITIQUE OF LOCALISATIONISM

*Das Symptom* offers a sophisticated examination of localizationism. Goldstein first critically reviews the nineteenth-century notion of neuro(psycho)logical symptoms, which he had previously accepted as a disciple of Carl Wernicke (1848-1905). At that time, symptoms were conceived as regularly occurring, monolithic phenomena believed to reveal the brain’s nature as a compilation of intertwined mechanisms. These mechanisms were supposedly identified through clinical examination as related to damaged brain areas, regarded as the “seats” of the respective functions. Each finding was thought to add a piece to the puzzle of brain functioning. According to Wernicke’s account of aphasia, for example, impaired “motor images” of words are located in the left inferior frontal gyrus (Broca’s area), leading to motor aphasia, while impaired “sound images” of words in the left superior temporal gyrus (Wernicke’s area) cause sensory aphasia. Fiber connections between these areas serve as transfer channels of sound images sent to motor images causing conduction aphasia. Thus, disturbances may affect the two sites of language representation as well as the connections between them, accounting for different patterns of aphasic syndromes.

A central issue in *Das Symptom* concerns Goldstein’s rejection of the nineteenth-century mechanistic approach for both neurophysiological and conceptual reasons. This approach fails to explain how to understand and treat brain damage sequelae, as it ignores the distinction between symptom and function*.* This distinction was introduced by the Russian-Swiss neuropathologist Constantin von Monakow (1853–1930)^5^. As Monakow argued, symptoms should be regarded as manifestations of defects caused by brain lesions, but not as impairments of functions per se. Naming difficulties following a stroke, *e.g*., may be linked to damaged brain areas, but naming itself may not be impaired, as difficulties could result from disconnection between functionally relevant areas (the Monakow *diaschisis* effect^6^). Temporary dysfunction of brain regions may also be caused by lesions affecting inter-regional connections, thereby hampering information transfer. Von Monakow also claimed that symptoms are dynamic: they change both quantitatively and qualitatively over time, partly due to cerebral compensation. From Monakow’s critical assessment of localisationist approaches, Goldstein concluded that empirical and methodological problems of localisationism appeared insuperable.

## *DAS SYMPTOM*: AN EPISTEMOLOGICAL HALLMARK OF NEUROPSYCHOLOGY

*Das Symptom* complements von Monakow’s critique by demonstrating that symptoms are often intrinsically complex, not only regarding the type and evolution of a lesion, but also with respect to how they are examined. In other words, the concept of distinct cerebral centers with clear boundaries arises from a specific conceptual and clinical-practical framework. For the sake of illustration, Goldstein^1^ refers to the reflex as a key nineteenth-century paradigm for both clinical neurological examination and the localization of functions. Reflexes were defined as rigid, invariant, context-independent units of behavior and thus served as prime candidates for later “elementist” approaches to mental processes. The reflex became a leading explanatory concept for cerebral localization^7^ as well as for “elementist” models of the mind more broadly, as reflected in the influence of behaviorism on twentieth-century psychology^8^. Accordingly, the notion of the reflex plays an important role in *Das Sympto*m. It serves to illustrate the epistemological split that is central to Goldstein’s *oeuvre*: while he attributes significant clinical value to reflexes, *e.g*., to distinguish between spinal and pyramidal damage, he disputes the mechanistic claim that reflexes constitute context-independent data in healthy individuals. He cites clinical evidence indicating that reflex intensity varies depending on numerous contextual factors, such as the position of the tested limb, the posture of the entire organism, the simultaneity of other movements, and shifts of attention toward or away from the tested limb. To minimize such confounding influences, neurologists were prompted to define standard testing procedures for each reflex, thereby isolating it from the cerebral context, from other activities of the organism, and from environmental conditions. While this common practice remains clinically relevant, the reflex as thus examined constitutes an artificial phenomenon with high diagnostic but limited theoretical value. Therefore, reflexes are not suitable as foundational elements in a neuropsychological theory aimed at explaining how the brain (and the organism as a whole) functions in real-life situations.

To support his approach, Goldstein also draws on a finding closely related to psychology, namely, the work of his colleague Wilhelm Fuchs^9^ on the phenomenon of pseudofovea*.* In a series of experiments on a patient with homonymous hemianopia, Fuchs demonstrated that the area of optimal acuity, as experienced by the subject, was not to be found at the edge of the non-impaired visual field (as one would expect), but dynamically shifted toward the center of the remaining field, where it was surrounded by a periphery of decreasing sharpness, resembling the pattern typical of normal vision. According to Goldstein^1^, the pseudofovea represents an organism’s inherent capacity to adapt to damage in ways that best preserve mental and biological continuity.

**Figure 1:**
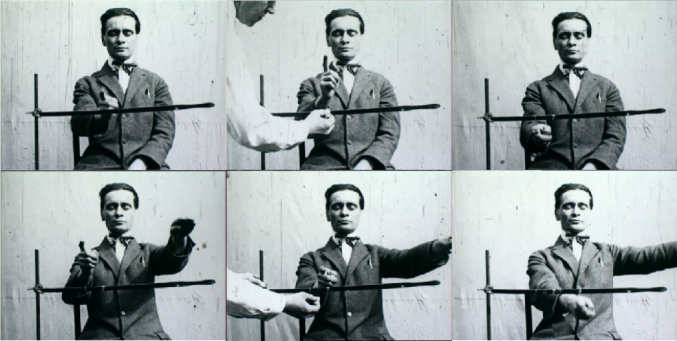
Kurt Goldstein examining a patient with a cerebellar lesion. The patient is sitting in front of a horizontally arranged bar. He is asked to move his right index finger repeatedly and consistently to a point in front of him, with his eyes closed. Goldstein (left) is marking the positions of the patient’s finger on the bar with chalk. Interestingly, the patient’s hand progressively drifts to the right. When the patient is instructed to perform the task while simultaneously lifting his left arm, the pointing movement of the right arm/index finger progressively shifts in the opposite direction, that is, to the left. Goldstein demonstrates that symptoms are not fixed but depend on the context of the whole organism. Stills taken from a didactic film recorded by Goldstein to illustrate his organismic conceptions^11,12^. With kind permission of the Rare Book and Manuscript Library at Columbia University, New York.

Mapping function to anatomy is further complicated by the fact that symptoms are never directly caused by lesions. Goldstein^1^ (p. 84, our translation) famously states: “[…] symptoms are an organism’s responses to specific questions raised by us.” In other words, symptoms are shaped by the way in which they are investigated^10^ (Figure 1). In sum, symptoms:

- result from each organism’s ability to cope with real-world demands in the aftermath of brain damage;
- are shaped by the means and practices of how they are isolated from a complex context (organism-environment).

As *signs* they do not stand on their own, but require interpretation, depending on the context in which they occur and the intentions of the examiner. In contrast to the on-off representations posited by mechanistic models, Goldstein^1^ (p. 96f) describes the brain as a single, global, and continuously active *Netzwerk* (network). Stimuli may cause shifts in activation, leading some brain regions to become more engaged than others, thereby enabling conscious representations or motor actions. Drawing on concepts from *Gestalt Psychology*, it could be said that lesions alter the foreground-background distinctiveness of neural activations, resulting in unstable, undifferentiated, primitive, and inflexible stimulus processing^1,10^. This occurs as *Grundfunktionen* (general abilities) are impaired, leading to a *Grundstörung* (basic disturbance).

## EVALUATION OF LURIA’S APPRAISAL OF *DAS SYMPTOM*

Why was *Das Symptom* so important for Luria to call it the founding document of neuropsychology? This is particularly striking given his assertion at beginning of his article^2^ that “[…] I cannot agree with most of [Goldstein’s] philosophical and theoretical ideas” (p. 311), and his criticism that Goldstein had failed to truly reconcile localizationist and holistic traditions in neuropsychology. How does this square with Luria’s^13^ conclusion, formulated around the same time, that Goldstein was but a proponent of the “noetic*”* school in neuropsychology (p. 19), which held that brain lesions result in the loss of general abilities (such as the capacity for abstraction^14^), rather than specific functions? Why, then, did *Das Symptom* count for Luria as starting point, especially considering that many of the contributions which Luria^2^ claimed followed this article, in fact preceded it, including “[…] the psychological qualification of the symptom” (p. 312)? It is worth noting that several of the ideas expressed in *Das Symptom* had already been developed in earlier articles by Goldstein^15,16^ and in texts published around the same time^17^. A survey of these contributions suggests that *Das Symptom* stands out primarily because it provides a more concise and comprehensive summary of the author’s central ideas, grounding them firmly in the Gestalt psychological foreground-background metaphor and the concept of *Grundstörung* (basic disturbance). Apart from that, however, *Das Symptom* does not differ significantly from Goldstein’s other conceptual papers of the period. It remains unclear how many of Goldstein’s publications Luria was able to access. From a correspondence between the two scholars in the mid-1930s, it is known that Luria deeply appreciated Goldstein’s pioneering work, especially his insistence on the qualitative analysis of symptoms, which Luria himself repeatedly emphasized in his own masterpiece, *Higher Cortical Functions in Man* ^13^. He had attempted several times to invite Goldstein to Moscow for discussions on functional localization and had even suggested translating some of Goldstein’s papers into Russian^18^. During the Pavlov affair^19^ shortly before Stalin’s death, Luria was severely criticized for having cited the work of the ‘bourgeois’ and ‘idealist’ Goldstein.

Although Luria^2^ was arguably justified in pointing out that Goldstein’s notion of *Grundstörung* was too coarse-grained for clinical use, he seems to have missed the core of Goldstein’s epistemological argument. This is further corroborated by passages in *Higher Cortical Functions in Man* ^13^, where Luria refers to Goldstein as a proponent of equipotentialism (p. 20) — the idea that functions are evenly distributed across the cortex. Yet Luria seemed to overlook the fact that Goldstein^20^ did not attempt to resolve the old localizationism-equipotentialism debate by merely amassing evidence in support of one side or the other. Rather, Goldstein aimed to transcend this binary opposition by exposing its methodological underpinnings and sought to integrate both perspectives at a higher level of analysis, where each could be considered informative from different perspectives. Although the holistic approach may appear more theoretically appealing, the idea of localization is neither conceptually incoherent nor clinically useless — it merely depends on specific conceptual and methodical assumptions. Knowledge of the brain and its functions does not derive from pieces of clinical and theoretical experience, but from the interaction between the person and his/her life-world, taken to be the primary perspective of clinicians. Luria^13^, in contradistinction, maintained that disturbances of higher cortical functions can be traced back to subfunctions with specific localization. To very briefly sum up convergences and divergences between Goldstein and Luria: Both agreed that symptoms do not mirror functions and that a psychological analysis of symptoms is indispensable for neuropsychology to succeed. They disagreed on the relevance of Goldstein’s distinction between concrete and abstract attitude and, most importantly with respect to the present contribution, they did not equally share the epistemological background. In *Das Symptom,* Goldstein emphasized the epistemological problems in identifying symptoms, as symptoms are useful but artificial phenomena, shaped by the investigator’s intentions and not representative of human organisms’ patterns of behavior and actions in their social and physical life-world.

It is unlikely that the actual beginning of neuropsychology will ever be clearly determined, but this question is secondary. It seems more rewarding to recognize the many implications of *Das Symptom*, even for contemporary neuroscience. There may be a temptation to discard Goldstein’s analyses as traces of the past, or perhaps as outdated compared to contemporary techniques of cerebral localization (such as functional MRI). As far as Goldstein is concerned, he conceived of cerebral localization as a conceptual issue, which did not depend entirely on the precision of localization. By way of analogy with the notion of clinical symptom, Goldstein might have argued that all that is achieved when localizing cerebral areas in action in fMRI is an ensemble of experimental effects rather than functions^20^. Experimental effects are as intrinsically complex as symptoms. They depend upon a variety of specific factors, such as the experimenter’s intentions, experimental designs, statistical procedures, and engineering implementation, to name just a few^21^. Thus, experimental effects are created by artificially isolating them from the organismic context. Goldstein’s great achievement, also in *Das Symptom*, consisted of thinking in new ways about brain research: cerebral functions may be analyzed, but only by intentionally abstracting them from the organismic context. This context is not only constituted by the living organism with an embedded brain, but also by the interactions of an investigator or clinician with his or her subject(s) and/or patient(s). If the context changes, the outlook of symptoms changes. The more fine-grained the analyses of cerebral processes are structured (*i.e*. down to the neuronal or even molecular level), the more we abstract by way of neglecting variables of interaction and by oversimplifying phenomena for the sake of investigation. Nature is thereby turned into something apt from which to receive appropriate answers that fit the questions^21^. *Das Symptom* was appreciated by Luria for good reasons, but the actual scope of its methodological implications seems to have gone unnoticed. Goldstein offered a potential for critical reflection that is still rarely taken seriously, even in today’s discussions on neuroscientific methodologies^20^.

## Data Availability

No empirical data were used for the present study.
